# The effect of unstable job on employee's turnover intention: The importance of coaching leadership

**DOI:** 10.3389/fpubh.2023.1068293

**Published:** 2023-03-16

**Authors:** Jeyong Jung, Byung-Jik Kim, Min-Jik Kim

**Affiliations:** ^1^Department of Police Science, University of Ulsan, Ulsan, Republic of Korea; ^2^College of Business, University of Ulsan, Ulsan, Republic of Korea; ^3^Department of Psychology, Yonsei University, Seoul, Republic of Korea; ^4^School of Industrial Management, Korea University of Technology and Education, Cheonan, South Chungcheong, Republic of Korea

**Keywords:** job insecurity, turnover intention, meaningfulness of work, coaching leadership, moderated mediation model

## Abstract

Swift social and economic environmental changes such as COVID-19 pandemic have led to increased job insecurity. The current study examines the intermediating mechanism (i.e., mediator) and its contingent factor (i.e., moderator) in the association between job insecurity and employee's turnover intention, especially from the perspective of positive psychology. By establishing a moderated mediation model, this research hypothesizes that the degree of employee meaningfulness in work may mediate the relationship between job insecurity and turnover intention. In addition, coaching leadership may play a buffering role to positively moderate the harmful impact of job insecurity on meaningfulness of work. With three-wave time-lagged data that was collected from 372 employees in South Korean organizations, the current study not only demonstrated that meaningfulness of work mediates the job insecurity–turnover intention relationship, but also that coaching leadership functions as a buffering factor in reducing the harmful influence of job insecurity on meaningfulness of work. The results of this research suggest that the level of meaningfulness of work (as a mediator) as well as coaching leadership (as a moderator) are the underlying processes and the contingent factor in the job insecurity–turnover intention link.

## Introduction

The COVID-19 pandemic caused a significant global shock that has potentially resulted in recession and economic crisis, leading to many employees around the globe losing their jobs (1). To survive this unexpected crisis, many companies have implemented massive restructuring and downsizing. As a result, employees have suffered from an increased sense of job insecurity (2–4). Job insecurity can be defined as “the perceived threat of losing the current job in the near future” [(5), p. 65].

Previous studies have demonstrated that job insecurity is closely associated with organizational outcomes. By functioning as a severe job stressor, job insecurity has been known to significantly predict employee's poor mental/physical health, burnout, job stress, turnover intention, decreased organizational commitment/identification/trust, work engagement, creativity, and organizational citizenship behavior (3, 6–15).

Although a number of studies on job insecurity have revealed the influence of job insecurity on several important organizational outcomes, we believe that there may still be some research gaps to be examined (8, 12).

First, previous scholars have suggested there is an inconclusive link between job insecurity and organizational outcomes (8, 12, 16). Specifically, several review papers (8, 12, 16) reported that job insecurity significantly diminishes the quality of individual-level, team-level, and organizational-level outcomes. The detrimental influences are because job insecurity tends to significantly elevate the degree of employee stress and negative responses such as perceptions, attitudes, and behaviors (6, 11, 16). By contrast, other scholars have demonstrated that job insecurity is likely to promote the quality of various outcomes or performances (13, 17, 18). Based on the job-preservation motivation perspective (13, 18), These results are derived from the understanding that an unstable job would urge employees to do their best to survive in their role by achieving high levels of performance. In addition, other research has reported that job insecurity is not associated with their outcomes in an organization (19). These varied and inconclusive results are due to a lack of research on intermediating processes (i.e., mediators) and contingent/contextual factors (i.e., moderators) in the association (12, 16). Therefore, our attempt to investigate various mediators and moderators is meaningful.

Second, extant works have paid relatively less attention to employee's positive psychology–associated mediators or moderators (including meaningfulness of work, forgiveness, gratitude, and coaching leadership) when explaining the underlying processes and contextual factors between job insecurity and organizational outcomes (8, 12, 20). In other words, those studies have mainly focused on “negative” aspects of organizational life.

Positive psychology has tried to explain a variety of organizational phenomena from the perspective of positive processes, attributes, and outcomes instead of negative ones (20). For example, previous scholars have reported that various negative variables such as threat to manifest/latent benefits of work (5), frustration of psychological needs (15), psychological contract breach (5), and injustice (21) function as critical mediators in the job insecurity–organizational outcomes relationship (8, 12). We acknowledge that negative mediators and moderators can meaningfully explain the influence of job insecurity in an organization. However, given that “real” organizational life includes both positive and negative aspects, examining the underlying mechanisms and its contingent factors from the perspective of positive psychology is required (8, 12, 22). This is the reason why the positive psychological approach has been acknowledged to possess theoretical and practical value (20).

Third, existing studies on job insecurity have underexplored the important role of leadership in the context of unstable job (8, 12, 16). Although scholars have reported a variety of boundary conditions (or contextual factors) that buffer the negative influence of job insecurity, they have mainly focused on individual-level variables including self-esteem, self-efficacy, internal locus of control, proactive personality, psychological capital, resilience, job control, and emotional intelligence; or macro-level factors including labor market insecurity, social safety networks, and macro-economic conditions (23–32). Thus, few studies have examined the moderating role of leadership (8, 12, 16). Leaders significantly influence employees' perceptions, attitudes, and behaviors by assigning tasks, evaluating them, establishing (explicit and implicit) norms within an organization (33, 34). They are also regarded as a main actor symbolizing the organization itself from the perspective of employees (35). Thus, investigating the moderating role of leadership is meaningful.

To address the described research gaps, we explore the intermediating mechanism and its contextual factor in the relationship between job insecurity and turnover intention. This concept is defined as the degree to which a member wants to leave their current job or organization to seek another one (36, 37). More specifically, this research suggests that employee's meaningfulness of work mediates the association between job insecurity and turnover intention. Furthermore, coaching leadership may buffer the harmful impact of job insecurity on meaningfulness of work by positively moderating the relationship.

To empirically test our hypotheses, we present a moderated mediation model in this paper that uses structural equation modeling (SEM) with 3-wave time-lagged data from 372 Korean workers. We expect the findings of this study will contribute to both job insecurity and turnover intention literature as follows. First, in the current paper, we try to elucidate the inconclusive relationship between job insecurity and organizational outcomes by exploring the intermediating mechanism (i.e., a mediator) and its contextual factor (i.e., a moderator) of the relationship. Second, we explore the intermediating process and its contingent variable from the perspective of positive psychology (i.e., meaningfulness of work and coaching leadership). Third, the research emphasizes the important role of leadership by demonstrating that coaching leadership, as one of the emerging leadership styles, functions as a buffering factor that diminishes the harmful effect of job insecurity on meaningfulness of work. Lastly, from a methodological point of view, this study complements the limitations of cross-sectional data by applying a longitudinal approach (i.e., 3-wave time-lagged research design).

## Theories and hypotheses

### Job insecurity and turnover intention

In the current research, we propose that job insecurity would functions as a critical antecedent of employee turnover intention (8, 36, 38). According to the conservation of resources theory (38), when an individual copes with the threat of resource loss, they may seek to replace resources. Thus, when an employee feels a sense of threat of resource due to instability in their job, they are likely to redirect their energies and resources away from their current role to search for new and more stable employment (8, 38). Based on this argument, this research anticipates that job insecurity will be shown to increase employee turnover intention.

**Hypothesis 1:** An employee's job insecurity may increase their turnover intention.

### Job insecurity and meaningfulness of work

We suggest that job insecurity will reduce the degree of employee's meaningfulness of the work. Meaningfulness of work is defined as the general beliefs, values, and attitudes that employees have about their work (39) as well as the degree to which employees consider their work to be valuable and important (40). Existing studies have considered that the perception of meaningfulness of work is rooted in the subjective interpretation of each employee's experiences and interactions at work (40–42). Being based on self-efficacy perspective (43, 44), those studies reveal that employees' self-efficacy, self-esteem, and competence are the essential antecedents of their meaningfulness of work (41, 45, 46). An employee who not only successfully completes assigned tasks but also effectively makes positive changes in an organization would feel a sense of self-efficacy and self-esteem. Then, they are likely to experience a high level of meaningfulness of work (46, 47). However, in a state of job insecurity, employees feel great psychological stress, anxiety, and exhaustion, which significantly diminishes their self-esteem, self-confidence, and self-efficacy (48–51). Those negative psychological effects substantially reduce their sense of meaningfulness of work. Based on the arguments, we suggest this hypothesis.

**Hypothesis 2:** An employee's job insecurity may reduce their meaningfulness of work.

### Meaningfulness of work and turnover intention

In the current study, we propose that diminished employee meaningfulness of work will increase their turnover intention. An employee tends to want their work to be more than just a means of making money; thus, they attempt to search for meaning in the workplace (41, 52). Extant studies have reported that employees' meaningfulness of work is likely to enhance their positive perceptions and attitudes, such as job satisfaction, organizational commitment, and intrinsic motivation (40, 46, 53–55), eventually enhancing positive emotions and their psychological states (56).

To be specific, the influence of meaningfulness of work on turnover intention can be explained by social exchange theory (57, 58). According to the social exchange approach, an individual or a group is likely to keep balance in relationships, that is called “the rule of reciprocity” (58, 59). When an individual or a group is provided something by someone or group, the beneficiary would perceive a sense of duty to repay it similarly (57, 59). For example, from the perspective of employees, the aforementioned positive or negative psychological states which are originated in meaningfulness of work may be perceived as “additional rewards” beyond an official contract (46, 54) since the employees receive a monetary reward for their labor. Then, the employees are likely to perceive a sense of obligation to repay the additional rewards to their organization. To repay it, the employees are likely to show positive attitudes toward their organization. Then, those positive inner states and experiences reduce the employee's degree of intention to leave their organization. However, if the employee feels a sense of job insecurity, they do not experience positive psychological states, even suffering from negative emotions such as anxiety, depression, and anger. Then, their turnover intention is increased. Taken together, we propose the following hypothesis.

**Hypothesis 3:** Decreased employees' meaningfulness of work may increase their turnover intention.

### The mediating role of meaningfulness of work

Based on the above arguments, and to integrate the relationships between the research variables in the mediation structure (i.e., job insecurity, meaningfulness of work, and turnover intention), we suggest that meaningfulness of work will mediate the relationship between job insecurity and turnover intention. From the perspective of positive psychology, an employee's positivity (such as meaningfulness of work) may not only reduce due to negative perceptions such as job insecurity but may also directly reduce their negativity, such as turnover intention.

**Hypothesis 4:** Employees' meaningfulness of work may mediate the relationship between job insecurity and turnover intention.

### The moderating role of coaching leadership

Furthermore, and more importantly, this research suggests that coaching leadership functions as a critical moderator to buffer the negative impact of job insecurity on meaningfulness of work. As already stated, our argument that job insecurity diminishes the level of employee meaningfulness of work may be reasonable and acceptable. However, the impact of job insecurity on meaningfulness of work may not always be valid in all situations or contexts in the same way because there are several contextual/contingent factors (such as personality, gender, age, leadership style, organizational climate, rule, and systems) that positively/negatively moderate the job insecurity–meaningfulness of work relationship in a real organization. Among several potential moderators, we focus on the role of leadership as a leader not only significantly influences employees' perceptions and attitudes by assigning tasks, evaluating the results, and establishing rules (34), but is also regarded as the main actor who symbolizes the organization itself from the employees' point of view (35).

Among various leadership styles, this paper focuses on coaching leadership. This concept can be defined as a leaders' behaviors that help followers effectively solve and cope with problems, difficulties, and conflicts in an organization, thereby enhancing their performance and helping them fully realize their potential and growth (60, 61). According to Heslin et al. (62), coaching leadership consists of three factors: (1) guidance, (2) facilitation, and (3) inspiration. First, guidance means providing constructive and positive feedback to followers on specific organizational expectations and goals and how to achieve them. Second, facilitation refers to helping followers analyze and explore how to solve job-related problems and improve their performance on their own. Third, inspiration means helping followers recognize their potential and value, motivating them to achieve better performance. Through these three factors, followers can develop their potential and capabilities to achieve greater self-efficacy and competence (60, 61). With increased coaching leadership, employees feel more respect and support from their leader (62, 63). This enables followers to develop a positive self-concept within the organization, feeling respected by the organization. Previous studies have reported that coaching leadership is closely and positively associated with employees' psychological wellbeing, job satisfaction, work performance, and organizational citizenship behavior (61–67).

In this paper, we propose that coaching leadership mitigates the detrimental influence of job insecurity on meaningfulness of work. A leader's coaching behaviors provide effective guidance for their followers to adequately deal with negative emotional states, personal problems, and crises that originate from job instability (62). This leadership enables followers to feel a sense of respect, mutual trust, support, and self-worth within the organization, eventually reducing the negative effects of job insecurity on meaningfulness of work (60, 68). For example, when the level of coaching leadership is high, a leader's coaching behaviors guide employees to effectively cope with anxiety and fear from unstable employment, even if they feel a high degree of job insecurity. As a result, followers are less likely to feel diminished levels of meaningfulness of work.

**Figure 1:**
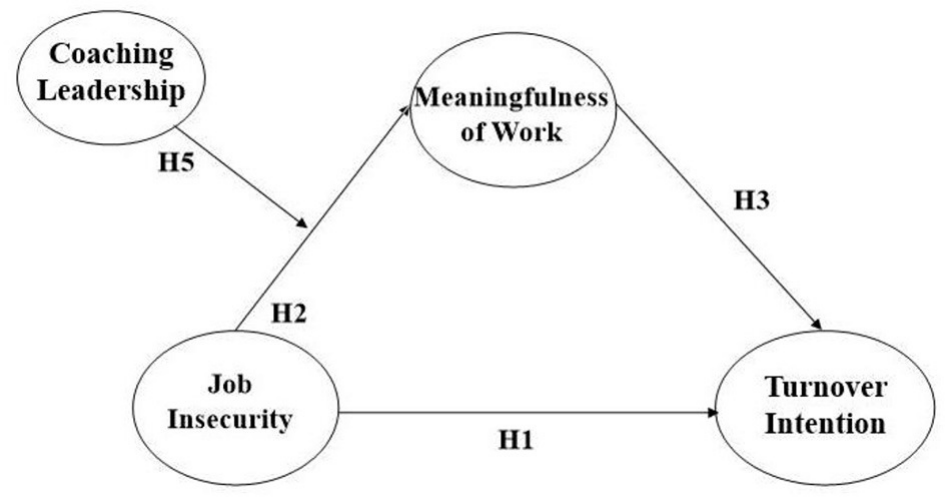
Theoretical model.

By contrast, when the level of coaching leadership is low, followers may experience difficulty dealing with negative emotions, personal problems, and crises that are derived from unstable employment, making them feel less respected and supported by the organization (62, 64). Therefore, less coaching leadership will make employees who suffer from job insecurity perceive that they cannot adequately address the problem and are isolated from the organization. As a result, the negative impact of job insecurity may not be properly resolved and may even be amplified. Thus, we suggest the following hypothesis (please see Figure 1).

**Hypothesis 5:** Coaching leadership may positively moderate the relationship between job insecurity and meaningfulness of work by reducing the negative effect of job insecurity on meaningfulness of work.

## Methods

### Participants and procedure

The sample consisted of currently working employees over 19 years old in various organizations in South Korea across three different time points. They were recruited through an online survey company which has an online survey system with the largest population of research panelists of ~3,450,000. The participants reported their occupational status when they registered for online membership *via* a user authentication system (i.e., cellular phone number or email address). Such online survey systems are considered a reliable method for accessing various samples (69).

Data was collected from employees of South Korean firms at three different time points. This was done in an effort to complement the fundamental issue embedded in cross-sectional research design. The research company randomly provided an identification number for each respondent. And the identification number was managed through the online surveying system of the company. Based on this method, we could match the questionnaires at the 3 time points. The operating function of the online system allowed us to track who responded to our survey, confirming that participants from time point 1 to time point 3 were the same. The interval between each stage was 4–5 weeks. Our survey system was open for 2 or 3 days each at each time point to provide enough time for participants to respond. When the system was open, participants could access it at any time. The company monitored the integrity of data by using traps for geo-IP violators and timestamps to flag efficient response time, which restricted participants from logging into the survey site and filling out the surveys multiple times.

The research firm contacted the participants directly to establish consent to participate in the survey, ensuring not only that their participation would be voluntary but also that their responses would be confidential and only used for research purposes. The company also reported and obtained informed consent and compliance with ethical requirements from those who agreed with the participation and reporting. The company provided the participants with a reward for their participation in the form of cash (US $8). The study was approved by the Institutional Review Board of a representative university in South Korea.

The research company selected the participants with a random stratified selection process to reduce the possibility of sampling bias. In stratified sampling, a random sample is drawn from each of the requisite strata. Through this sampling method, the possibility of bias from various employee characteristics that may influence the results of this research (e.g., gender, age, position, education, and industry type) is reduced. Due to its various operating functions in the online systems, this paper was able to track down who responded to it, implying that respondents from time point one to time point three are the same.

During time point 1, 512 employees participated in our survey, with 421 taking part at time point 2, and 379 at time point 3. After collecting the data, we eliminated responses with missing data. Finally, the study utilized data from 372 employees who provided complete answers to all three waves of the survey (response rate: 72.66%). To determine the sample size, we utilized various suggestions from previous research. First, we checked whether our sample size was appropriate by calculating the minimum sample size with G^*^Power version 3.1.9.7. Power analysis demonstrated that a sample size of 372 provided sufficient power (≥0.80) to detect a medium effect with an alpha level of *p* = 0.05 (70). In addition, Barclay et al. (71) suggest that one observable variable needs at least 10 cases (i.e., the rule of 10) when conducting SEM. Because the research model in this study comprised 22 observable variables, our final sample of 372 cases was considered an adequate sample. The characteristics of the respondents are described in Table 1.

**Table 1:** Descriptive characteristics of the sample.

**Characteristic**	**Percent**
**Gender**	
Male	50.8%
Female	49.2%
**Age (years)**	
20–29	14.0%
30–39	36.5%
40–49	33.1%
50–59	16.4%
**Education**	
Below high school	8.6%
Community college	19.4%
Bachelor's degree	60.5%
Master's degree or higher	11.6%
**Occupation**	
Office worker	71.3%
Profession (practitioner)	7.2%
Public official	6.0%
Manufacturing	5.7%
Sales and marketing	4.3%
Administrative positions	4.0%
Education	0.3%
Others	1.2%
**Position**	
Staff	23.1%
Assistant manager	22.6%
Manager or deputy general manager	32.8%
Department/general manager or director and above	21.5%
**Tenure (years)**	
Below 5	47.0%
5–10	27.2%
11–15	12.9%
16–20	7.0%
21–25	2.4%
Above 26	3.5%
**Industry type**	
Manufacturing	24.3%
Wholesale/retail business	12.5%
Construction	12.9%
Health and welfare	9.9%
Information services and telecommunications	8.6%
Education	8.1%
Services	6.5%
Financial/insurance	3.8%
Consulting and advertising	1.3%
Others	11.3%

### Measures

At each time point, the survey measured distinct variables in our research model. At time point 1, the respondents were asked about the level of job insecurity and coaching leadership. At time point 2, participants' data were collected to measure their degree of meaningfulness of work. At time point 3, data were collected on participants' turnover intention. These variables were assessed through multi-item scales on a 5-point Likert scale (1 = *strongly disagree*, 5 = *strongly agree*). Moreover, through Cronbach alpha values, the internal consistency of each variable was computed.

### Job insecurity (time point 1, collected from members in an organization)

We used four items for the job insecurity scale (72). Sample items were: “*If my current organization were facing economic problems, my job would be the first to go*,” “*I will not be able to keep my present job as long as I wish*,” and “*My job is not a secure one*.” The Cronbach's alpha value was 0.90.

### Coaching leadership (time point 1, collected from employees)

To measure the degree of coaching leadership, we utilized 12 items from previous studies on coaching leadership (60, 61). Sample items were: “*My leader believes in my potential for growth*,” and “*My leader asks questions that make me reflect on my thoughts and perspectives*.” The Cronbach's alpha value was 0.94.

### Meaningfulness of work (time point 2, collected from employees)

To measure the level of employee meaningfulness of work, the current study used five items of the meaningfulness of work scale from extant works (73, 74). Sample items were: (a) “*The work that I do is meaningful*”; (b) “*The work that I do makes the world a better place*”; and (c) “*My work is one of the most important things in my life*.” The Cronbach's alpha value was 0.88.

### Turnover intention (time point 3, collected from employees)

The degree of turnover intention was measured through three items from existing studies (36, 37). The items were: (a) “*How likely is it that you will look for a job outside of this organization during the next year*?” (b) “*How often do you think about quitting your job at this organization*?” and (c) “*If it were possible, how much would you like to get a new job*?” The Cronbach's alpha value was 0.89.

### Control variables

Based on extant studies (36, 37), the dependent variable of this research—turnover intention—was controlled by various factors such as tenure, gender, position, and education of an employee. The control variables were collected at time point 2.

### Statistical analysis

First, frequency analysis was performed to check the participants' demographic features. We conducted Pearson correlation analysis using the SPSS 26 program to assess the relationships between our research variables. Then, following the suggestion of Anderson and Gerbing (75), we took a two-step approach that consists first of measurement and then the structural model. To test the validity of the measurement model, we performed confirmatory factor analysis (CFA). Next, based on SEM, a moderated mediation model analysis with the maximum likelihood (ML) estimator was performed using the AMOS 23 program to test the structural model.

To test whether various model fit indexes are acceptable, this study utilized a variety of goodness-of-fit indices including the comparative fit index (CFI), the Tucker–Lewis index (TLI), and the root mean square error of approximation (RMSEA). Extant research has reported that the CFI and TLI values >0.90 and an RMSEA value of < 0.06 are appropriate (76). Finally, bootstrapping analysis was implemented to test whether the indirect effect was significant (77). Lastly, to check whether our mediation hypothesis was supported, we conducted bootstrapping analysis with a 95% bias-corrected confidence interval (CI). This analysis can check the significance of the indirect mediation effect. If the CI does not include zero (0), this result indicates that the indirect effect is statistically significant with a 0.05 level (77).

## Results

### Descriptive statistics

Research variables (job insecurity, coaching leadership, meaningfulness of work, and turnover intention) were significantly related. The correlation analysis results are shown in Table 2.

**Table 2:** Correlation between research variables.

	**Mean**	**S.D**.	**1**	**2**	**3**	**4**	**5**	**6**	**7**
1. Gender_T2	1.49	0.50	–						
2. Education_T2	2.75	0.77	−0.15^**^	–					
3. Tenure_T2	7.45	7.29	−0.27^**^	0.01	–				
4. Position_T2	2.92	1.59	−0.42^**^	0.22^**^	0.29^**^	–			
5. Job insecurity_T1	2.82	0.85	−0.05	−0.05	0.01	0.11^*^	–		
6. CL_T1	3.16	0.73	−0.06	0.05	0.01	0.10^*^	−0.06	–	
7. MoW_T2	3.13	0.79	−0.19^**^	0.16^**^	0.14^**^	0.23^**^	−0.17^**^	0.29^**^	–
8. TI_T3	3.09	1.03	0.13^*^	0.04	−0.19^**^	−0.03	0.12^*^	−0.25^**^	−0.34^**^

^*^p < 0.05. ^**^p < 0.01. S.D., standard deviation; CL, coaching leadership; MoW, meaningfulness of work; TI, turnover intention. As for gender, males are coded as 1 and females as 2. As for position, general manager or higher are coded as 5, deputy general manager and department manager 4, assistant manager 3, clerk 2, and others below clerk as 1. As for education, “below high school diploma” level is coded as 1, “community college” level as 2, “bachelor's” level as 3, and “master's degree or more” level is coded as 5.

### Measurement model

To test the discriminant validity of the main research variables (job insecurity, coaching leadership, meaningfulness of work, and turnover intention), we performed CFA for all items by checking the measurement model's goodness-of-fit. To be specific, we compared our hypothesized model, a 4-factor model (job insecurity, coaching leadership, meaningfulness of work, and turnover intention), to other alternative models, such as 3-, 2-, and 1-factor models, by conducting a series of chi-square difference tests.

First, the hypothesized 4-factor model had a good and acceptable fit [$\chi_{\left(\text{df}=109\right)}^{2}$ = 212.224; CFI = 0.974; TLI = 0.967; RMSEA = 0.051]. Then, we conducted a series of chi-square difference tests by comparing the 4-factor model with a 3-factor model [$\chi_{\left(\text{df}=112\right)}^{2}$ = 1239.387; CFI = 0.715; TLI = 0.655; RMSEA = 0.165], a 2-factor model [$\chi_{\left(\text{df}=114\right)}^{2}$ = 1699.100; CFI = 0.600; TLI = 0.523; RMSEA = 0.194], and a 1-factor model [$\chi_{\left(\text{df}=115\right)}^{2}$ = 1763.298; CFI = 0.584; TLI = 0.508; RMSEA = 0.197]. The results of the chi-square difference tests showed that the 4-factor model was better than others. Thus, this result means that our four research variables have an appropriate degree of discriminant validity.

### Structural model

In this study, we built a moderated mediation model including both mediation and moderation structures in the job insecurity–turnover intention relationship. In the mediation structure, the job insecurity–turnover intention relationship is mediated by the degree of employee meaningfulness of work. In the moderation structure, coaching leadership functions as a buffering factor that positively moderates the harmful impact of job insecurity on meaningfulness of work.

Next, in the moderation structure, we multiplied the two variables (i.e., job insecurity and coaching leadership) to make an interaction term between the variables. Before the multiplication, the two variables were centered on their means to decrease the harmful impact of multicollinearity. Such a centering method increases the validity of the moderation analysis by diminishing the degree of multi-collinearity between the variables and minimizing the loss of correlations (78).

To test the impact of the multicollinearity bias, we measured the value of variance inflation factors (VIF) and tolerances (78). The VIF values for job insecurity and coaching leadership were 1.003 and 1.003, respectively. Moreover, the values of tolerance were 0.997 and 0.997, respectively. The results with VIF values smaller than 10 with the tolerance values above 0.2 indicate that job insecurity and coaching leadership are relatively free from the multi-collinearity issue.

### Results of mediation analysis

To find the best mediation model, we compared a full mediation model to a partial mediation model by performing a chi-square difference test. The full mediation model is identical to the partial mediation model except for the direct path from job insecurity to turnover intention. The fit indices of both the full mediation model [χ^2^ = 254.539 (df = 136), CFI = 0.962, TLI = 0.952, and RMSEA = 0.048] and the partial mediation model [χ^2^ = 253.441 (df = 135), CFI = 0.962, TLI = 0.952, and RMSEA = 0.049] were acceptable. However, the chi-square difference test between the models [Δ$\chi_{\left(1\right)}^{2}$ = 1.098, non-significant] demonstrated that the full mediation model was superior. This result indicates that job insecurity is likely to indirectly influence (e.g., *via* mediating effect of meaningfulness of work) turnover intention, rather than having a direct impact.

The control variables (tenure, gender, education, and position) were included in the research model to control for the dependent variable, turnover intention. The result showed that only position (β = 0.11, *p* < 0.05) and tenure (β = −0.18, *p* < 0.05) were statistically significant.

Including the control variables, our research model showed that job insecurity was non-significantly associated with employee's turnover intention (β = 0.06, *p* > 0.05), which does not support Hypothesis 1. For Hypothesis 1, the coefficient value of the path from job insecurity to turnover intention was in the “partial” mediation model (which was inferior to the full mediation model), not the full mediation model that was finally accepted. This result is consistent with the fact that the model fit indices of full mediation are better than partial mediation. Based on the results of the chi-square difference test between full and partial mediation models as well as the non-significant value of the path coefficient, we conclude that Hypothesis 1 was not supported. In other words, job insecurity is likely to influence turnover intention in an “indirect” way through the mediating effect of various mediators (e.g., meaningfulness of work) rather than in a direct way.

**Figure 2:**
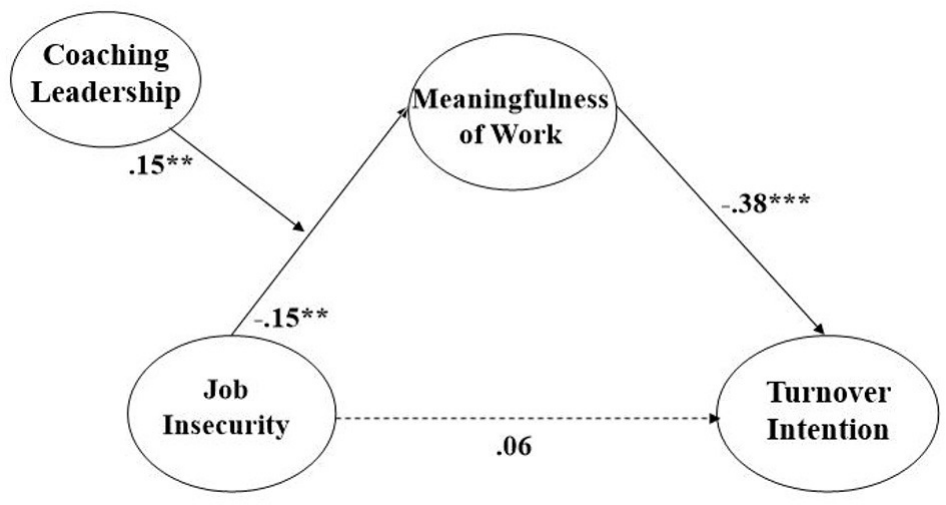
Coefficient values of our research model (^**^p < 0.01, ^***^p < 0.001. All values are standardized).

Job insecurity was significantly and negatively associated with employee's meaningfulness of work (β = −0.15, *p* < 0.01), supporting Hypothesis 2, and that meaningfulness of work is significantly and negatively associated with turnover intention (β = −0.38, *p* < 0.001), supporting Hypothesis 3 (please see Table 3 and Figure 2).

**Table 3:** Results of structural model.

**Hypothesis**	**Path (relationship)**	**Unstandardized estimate**	**S.E**.	**Standardized estimate**	**Supported**
1	Job insecurity -> turnover intention	0.067	0.062	0.058	No
2	Job insecurity -> meaningfulness of work	−0.124	0.047	−0.150^**^	Yes
3	Meaningfulness of work -> turnover intention	−0.522	0.080	−0.379^***^	Yes
5	Job insecurity × coaching leadership	0.157	0.056	0.151^**^	Yes

^**^p < 0.01. ^***^p < 0.05. Estimate indicates standardized coefficients. S.E., standard error. The coefficient value of the path from job insecurity to turnover intention (H1) was in the partial mediation model which was not accepted as a final model.

### Bootstrapping

To test the mediation effect of meaningfulness of work in the job insecurity–turnover intention relationship (Hypothesis 4), we conducted bootstrapping analysis with a sample of 10,000 (77). The indirect mediation effect would be significant at a 5% level if the 95% bias-corrected CI for the effect of mean indirect mediation excluded 0 (77).

The results showed that the bias-corrected CI for the mean indirect effect did not include 0 [95% CI = (0.014, 0.109)]. This means that that the indirect mediation effect of meaningfulness of work was statistically significant, supporting Hypothesis 4. The direct, indirect, and total effects of the paths from job insecurity to turnover intention are shown in Table 4.

**Table 4:** Direct, indirect, and total effects of the final research model.

**Model (hypothesis 4)**	**Direct effect**	**Indirect effect**	**Total effect**
Job insecurity -> meaningfulness of work -> turnover intention	0.000	0.057	0.057

All values are standardized.

### Result of moderation analysis

**Figure 3:**
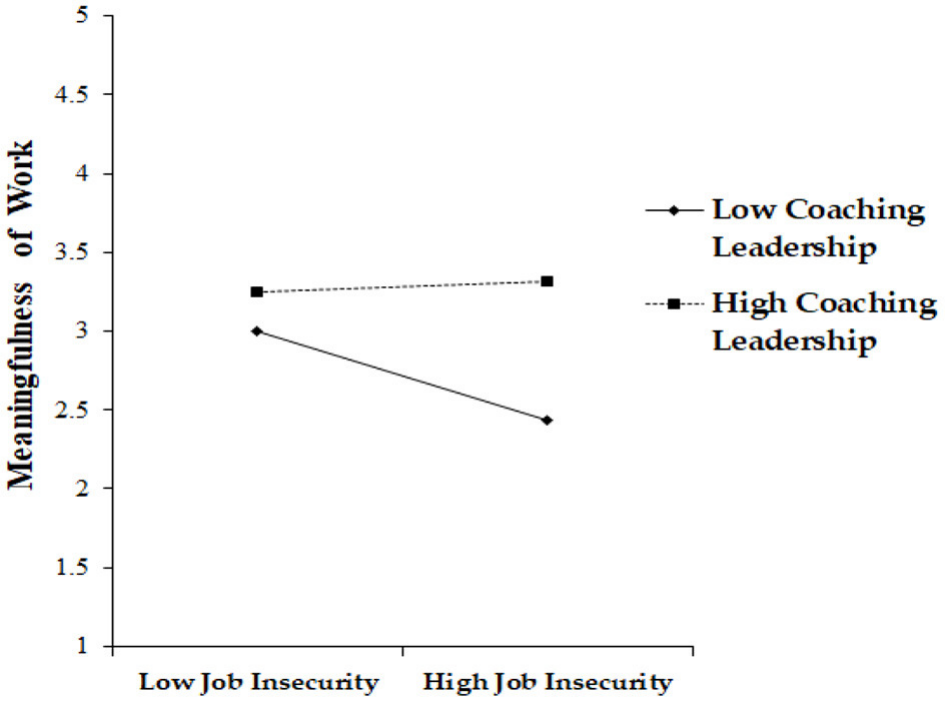
Moderating effect of coaching leadership in the job insecurity–meaningfulness of work link.

We tested the moderation effect of coaching leadership on the relationship between job insecurity and meaningfulness of work. To do so, we conducted a mean-centering process by making an interaction term. The coefficient value of the interaction term (β = 0.18, *p* < 0.001) was statistically significant. This result means that coaching leadership positively moderates the relationship between job insecurity and job stress by playing a buffering role. Moreover, it indicates that when the level of coaching leadership is high, the decreasing impact of job insecurity on meaningfulness of work is reduced, supporting Hypothesis 5 (please see Figure 3).

## Discussion

Utilizing 3-wave time-lagged data from 372 employees in South Korea, the current study demonstrated that an employee's meaningfulness of work functions as an important intermediating process (i.e., mediator) in the job insecurity–turnover intention relationship. Moreover, this research empirically determined that coaching leadership plays a buffering role that reduces the harmful influence of job insecurity on meaningfulness of work (i.e., moderator). These results are consistent with the arguments of previous works on job insecurity, meaningfulness of work, turnover intention, and coaching leadership. To be specific, our result that job insecurity functions as a critical antecedent of employee turnover intention is consistent with the extant studies [please see Jiang and Lavaysse (8)]. And our result that job insecurity decreases the degree of meaningfulness of work corresponds with the existing works (45, 46). Also, the result that the diminished meaningfulness of work increases the degree if turnover intention is also consistent with the previous works (52, 59). Lastly, the moderating effect of coaching leadership is consistent with the recent works (63, 67). The reason why the results are consistent with the previous work is that our arguments are based on the proper and validated theoretical background.

This paper may contribute to the literature on job insecurity, meaningfulness of work, turnover intention, and coaching leadership by revealing why (i.e., mediator) and when (i.e., moderator) job insecurity influences turnover intention. In the following sections, we describe the theoretical/practical implications and limitations of the study, also providing suggestions for future research.

### Theoretical implications

We believe that the current study may contribute to job insecurity literature from a theoretical perspective. First, the current paper may contribute to job insecurity literature by resolving the inconclusive relationship between job insecurity and organizational outcomes. To address the issue of inclusive results in the job insecurity-organizational outcomes link, we explored the intermediating process (i.e., mediators) and its contingent factor (i.e., moderator) of the relationship (12). As a result, we found and suggest that job insecurity negatively affects organizational outcomes *via* deteriorating employees' perceptions or attitudes at work (i.e., meaningfulness of work). To be specific, we unveiled the mediating role of employees' meaningfulness of work in the job insecurity–turnover intention link. Furthermore, by empirically validating that coaching leadership plays a buffering role in the relationship between job insecurity and meaningfulness of work, this research can shed light on a contextual or contingent factor which moderate the influence of job insecurity in an organization. We believe that this research can contribute to resolving the inconclusive results of job insecurity by bolstering existing studies that show a detrimental influence of job insecurity on organizational outcomes.

Second, this study attempts to interpret job insecurity from the perspective of positive psychology, by emphasizing the importance of a positive psychological intermediating mechanism (i.e., meaningfulness of work) and its contextual factor (i.e., coaching leadership). In other words, we believe that the current paper may contribute to expanding the scope of job insecurity literature by integrating job insecurity literature with positive psychology literature. In this paper, we demonstrated that an employee's meaningfulness of work, as a representative variable of positive psychology, plays a mediating role in explaining the impact of job insecurity on turnover intention. Thus, this paper reveals that job insecurity significantly diminishes employee positivity (i.e., meaningfulness of work), eventually leading to them leaving the organization. However, this paper also suggests that an employee's positivity in an organization can be recovered and protected through the positivity of the leader, for example, through coaching leadership. Positive leadership behavior can function as a buffering factor, which positively moderates the harmful effect of job insecurity on meaningfulness of work.

Third, the current study demonstrates that leadership plays a critical buffering role in explaining the negative impact of job insecurity. Considering that members in an organization tend to be influenced by their leaders' thoughts, feelings, words, and behaviors when they interpret the meaning of various events, systems, and situations around them (34, 35), leaders can substantially affect employees' perceptions and attitudes toward an important event or situation, such as job insecurity. In other words, leadership will significantly moderate the impact of job insecurity in an organization. Specifically, we show that the harmful influence of job insecurity on meaningfulness of work would be reduced by a high level of coaching leadership. When the level of coaching leadership is high, employees are likely to perceive that they are not alone and separated from their organization. Then, the negative impact of job insecurity can be alleviated. This result indicates that the coaching behaviors of a leader can be an important contingent variable in mitigating the harmful influences of job insecurity.

### Practical implications

The current study can provide practical contributions for top management teams who want to understand the influence of job insecurity. First, the result of this paper suggests that top management teams should understand the seriously harmful impact of job insecurity on employees' turnover intention. We empirically demonstrate that job insecurity significantly boosts employees' turnover intention. Given that an employee's turnover is likely to be closely associated with several organizational outcomes, job insecurity substantially diminishes the level of an organization's competitive advantage and sustainability. Therefore, in this paper, we propose that top management teams are required to address and resolve this critical issue carefully by establishing effective and efficient human resource management systems.

Second, we provide useful indicators or criteria (i.e., meaningfulness of work as a mediator in the research model) for top management teams in monitoring and checking the harmful influences of job insecurity as well as the effectiveness of several buffering variables (e.g., coaching leadership, and several practices for decreasing the harmful impacts of job insecurity). Our results empirically demonstrate that the degree of an employee's meaningfulness of work plays the role of mediator in the job insecurity–turnover intention relationship. This indicates that the degree of an employee's meaningfulness of work can be utilized as an important measure or criteria to evaluate how severely job insecurity affects employee turnover intention.

The buffering effect of coaching leadership can also be measured or evaluated by the change of employees' meaningfulness of work. For example, when the level of meaningfulness of work does not change after implementing coaching leadership in an organization, the top management teams may interpret that the buffering influence of coaching leadership will not work adequately. In summary, we propose in this paper that top management teams should monitor the degree of employee meaningfulness of work to check the influences of both job insecurity and its moderating variable (i.e., coaching leadership).

Third, the current study also provides direction for top management teams who attempt to diminish the negative influence of job insecurity in an organization. We suggest that top management teams should understand and properly utilize the positive and buffering effects of coaching leadership. To alleviate the harmful influence of job insecurity, top management teams should apply coaching leadership in their organization. By providing training for coaching leadership behaviors, top management teams can cultivate effective coaching leaders and a coaching culture within an organization. This would significantly contribute to addressing the negative influence of job insecurity.

### Limitations and suggestions for future research

Although we believe the current study may meaningfully contribute to job insecurity and turnover intention literature, there are still some limitations to be addressed. First, this research could not measure the level of job insecurity in an objective manner as the current study only utilized survey data relying on respondent's self-reporting, which would most likely be subjective. While we acknowledge that the objective phenomena (such as downsizing rate) may not directly influence employees' perceptions and attitudes because the objective characteristic (e.g., downsizing rate) tends to be interpreted through their sense-making processes, the objective measure would be unconsciously reflected in employees' responses. Thus, we suggest that future research needs to utilize both the subjective and objective measures and compare the differential effects of the different measures. Second, this research could not properly consider a number of external factors that substantially affect the degree of job insecurity. There are numerous objective factors that pervade an employee's perception of their subjective job insecurity, such as downsizing rates, the quality or characteristics of human resource management systems, and features of the social security system at the country level (9). Therefore, we suggest that future research should more fully consider the issue by elaborately controlling the objective variables.

Third, although the fundamental values and spirit of coaching leadership may be universal in Western and Eastern societies (79, 80), a number of cultural differences may exist with regard to understanding the role of leadership. These will eventually influence employees' responses toward leadership style. Given that South Korea has been affected by Confucian hierarchy for many centuries, Korean employees may be more familiar with the culture of command and discipline compared to the Western employees (79). As a result, Korean employees are likely to feel that a leader's coaching behaviors are not natural and effective in a real organization. Therefore, the results of the this study should be carefully interpreted.

## Conclusion

This research investigated the impact of job insecurity on employees' turnover intention. The results showed that job insecurity promotes the extent of employee turnover intention *via* the mediating role of meaningfulness of work. Moreover, coaching leadership functions as a positive moderator in the job insecurity–meaningfulness of work relationship. The results indicate that the level of employees' meaningfulness of work is an underlying mechanism in translating job insecurity into turnover intention. In addition, the degree of coaching leadership provides a buffering factor that decreases the negative influence of job insecurity. Although this research has some limitations, we anticipate that the findings will offer a positive contribution to expanding the literature on job insecurity.

## Data availability statement

The raw data supporting the conclusions of this article will be made available by the authors, without undue reservation.

## Ethics statement

The studies involving human participants were reviewed and approved by Macromill Embrain Group of Ethics Committee. Macromill Embrain Group is the company providing market research service and their approval is sufficient according to the local requirements. The patients/participants provided their written informed consent to participate in this study.

## Author contributions

JJ and B-JK contributed by writing the original draft of the manuscript and in the conceptualization, data collection, formal analysis, and methodology. M-JK contributed in the conceptualization, analysis, revision, and in editing the manuscript. All authors have read and agreed to the published version of the manuscript.
