# Corrigendum: The effect of unstable job on employee's turnover intention: the importance of coaching leadership

**DOI:** 10.3389/fpubh.2023.1200929

**Published:** 2023-05-31

**Authors:** Jeyong Jung, Byung-Jik Kim, Min-Jik Kim

**Affiliations:** ^1^Department of Police Science, University of Ulsan, Ulsan, Republic of Korea; ^2^College of Business, University of Ulsan, Ulsan, Republic of Korea; ^3^Department of Psychology, Yonsei University, Seoul, Republic of Korea; ^4^School of Industrial Management, Korea University of Technology and Education, Cheonan, South Chungcheong, Republic of Korea

**Keywords:** job insecurity, turnover intention, meaningfulness of work, coaching leadership, moderated mediation model

In the published article, there was an error in the **Funding** statement.

The article will no longer have a funding statement.

The authors apologize for this error and state that this does not change the scientific conclusions of the article in any way. The original article has been updated.

