# Supplementary material for: The effect of unstable job on employee's turnover intention: The importance of coaching leadership
Source: Front Public Health. 2023 Mar 16;11:1068293. doi: 10.3389/fpubh.2023.1068293 (PMC10060841; doi:10.3389/fpubh.2023.1068293)
Supplement: Supplementary file 1 [file Data_Sheet_1.pdf]

APPENDIX. Measures

1. Job Insecurity (Kraimer et al., 2005).

"If my current organization were facing economic problems, my job would be the first to go."

"I will not be able to keep my present job as long as I wish."

"My job is not a secure one."

"Regardless of economic conditions, I will not have a job at my current organization."

"My job will not be there although I want it."

2. Coaching Leadership (Ellinger et al., 2003; Huang & Hsieh, 2015).

"My leader asks questions that make me reflect on my thoughts and perspectives."

"When a situation needs my leader's experiences, he/she willingly discusses them."

"My leader believes in my potential for growth."

"When I work with my leader, he/she discusses his/her expectations with me."

"When I share my feelings with my leader, my leader appears to be comfortable."

"When a decision is to be made, my leader prefers to participate with others to determine the outcome"

"In discussion with me, my leader focuses on my individual needs."

"My leader views differences of opinion as constructive."

"In order to improve my performance, my leader serves as a role model."

"In facing conflict between individual needs and tasks, my leader puts priority on meeting people's needs."

"My leader appears to view learning and development as one of his/her major responsibilities."

"To improve work performance, my leader constantly provides feedback."

3. Meaningfulness of Work (Bunderson & Thompson, 2009; Wrzesniewski, McCauley, Rozin, Schwartz, 1997)

“The work that I do is meaningful”.

“The work that I do makes the world a better place”.

“My work is one of the most important things in my life”.

“I would choose my current work life again if I had the opportunity”.

“The work that I do is important”.

4. Turnover Intention of Employees (Tett & Meyer, 1993; Wright & Cropanzano, 1998).

“How likely is it that you will look for a job outside of this organization during the next year?”

“How often do you think about quitting your job at this organization?”

“If it were possible, how much would you like to get a new job?”
